# (*E*)-3-Dimethyl­amino-1-(4-pyrid­yl)prop-2-en-1-one

**DOI:** 10.1107/S1600536809030542

**Published:** 2009-08-08

**Authors:** Lei Ni, Guo-Wei Zhu, Hong Wei

**Affiliations:** a College of Chemistry and Biology, Beihua University, Jilin 132013, People’s Republic of China

## Abstract

The title compound, C_10_H_12_N_2_O, is approximately planar, the r.m.s. deviation of the non-H atoms from the mean plane being 0.099 Å.

## Related literature

For an isomer of the title compound with the same space group and similar unit-cell parameters, see: Ni *et al.* (2009 ▶).
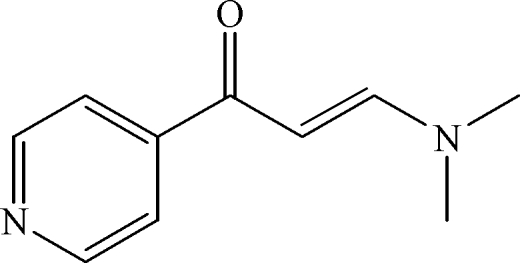


## Experimental

### 

#### Crystal data

C_10_H_12_N_2_O
*M* *_r_* = 176.22
Monoclinic, 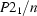

*a* = 5.6300 (11) Å
*b* = 22.850 (5) Å
*c* = 7.8400 (16) Å
β = 107.57 (3)°
*V* = 961.5 (3) Å^3^
*Z* = 4
Mo *K*α radiation
μ = 0.08 mm^−1^
*T* = 294 K
0.12 × 0.10 × 0.08 mm

#### Data collection

Bruker APEXII CCD diffractometer
Absorption correction: multi-scan (*SADABS*; Bruker, 2004 ▶) *T*_min_ = 0.990, *T*_max_ = 0.994
5177 measured reflections
1784 independent reflections
1503 reflections with *I* > 2σ(*I*)
*R*_int_ = 0.022

#### Refinement

*R*[*F* ^2^ > 2σ(*F* ^2^)] = 0.040
*wR*(*F* ^2^) = 0.137
*S* = 1.00
1784 reflections
121 parameters
H-atom parameters not refined
Δρ_max_ = 0.20 e Å^−3^
Δρ_min_ = −0.13 e Å^−3^

### 

Data collection: *APEX2* (Bruker, 2004 ▶); cell refinement: *SAINT-Plus* (Bruker, 2004 ▶); data reduction: *SAINT-Plus*; program(s) used to solve structure: *SHELXS97* (Sheldrick, 2008 ▶); program(s) used to refine structure: *SHELXL97* (Sheldrick, 2008 ▶); molecular graphics: *SHELXTL* (Sheldrick, 2008 ▶); software used to prepare material for publication: *SHELXTL*.

## Supplementary Material

Crystal structure: contains datablocks I, global. DOI: 10.1107/S1600536809030542/hb5024sup1.cif

Structure factors: contains datablocks I. DOI: 10.1107/S1600536809030542/hb5024Isup2.hkl

Additional supplementary materials:  crystallographic information; 3D view; checkCIF report

## Figures and Tables

**Fig. 1.**
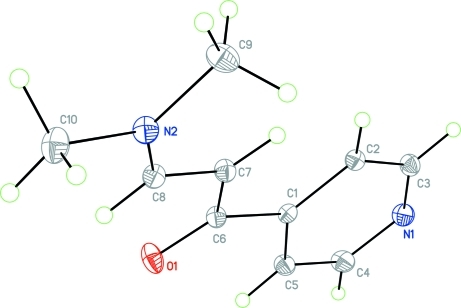
The molecular structure of (I), drawn with 30% probability displacement ellipsoids for the non-hydrogen atoms.

## supplementary crystallographic information

### Comment

As part of our ongoing studies of heteroaromatic compounds (Ni *et al.*,  2009), we now report the synthesis and structure of the title compound, (I).

As shown in Fig. 1, non-hydrogen atoms including the pyridine ring, *N*,*N*-Dimethylamino, and prop-2-en-1-one are coplanar with the r.m.s deviation of the fitted atoms being 0.099 Å.

### Experimental

A mixture of 4-acetylpyridine(10 mmol) and *N*,*N*-dimethylformamide-dimethyl acetal(40 ml) was refluxed for four hours. After concentration invacuo, recrystallization of the orange residue from ethanol afforded yellow blocks of (I). Anal. Calc. for C_10_H_12_N_2_O: C 68.10, H 6.81, N 15.89%; Found: C 68.05, H 6.69, N 15.81%.

### Refinement

All H atoms were geometrically positioned (C—H = 0.93–0.97Å) and refined as riding with *U*_iso_(H) = 1.2U_eq_(C) or 1.5U_eq_(methyl C).

### Crystal data

**Table:**

C_10_H_12_N_2_O	*F*(000) = 376
*M**_r_* = 176.22	*D*_x_ = 1.217 Mg m^−^^3^
Monoclinic, *P*2_1_/*n*	Mo *K*α radiation, λ = 0.71073 Å
Hall symbol: -P 2yn	Cell parameters from 1784 reflections
*a* = 5.6300 (11) Å	θ = 1.8–25.5°
*b* = 22.850 (5) Å	µ = 0.08 mm^−^^1^
*c* = 7.8400 (16) Å	*T* = 294 K
β = 107.57 (3)°	Block, yellow
*V* = 961.5 (3) Å^3^	0.12 × 0.10 × 0.08 mm
*Z* = 4	

### Data collection

**Table:**

Bruker APEXII CCD diffractometer	1784 independent reflections
Radiation source: fine-focus sealed tube	1503 reflections with *I* > 2σ(*I*)
graphite	*R*_int_ = 0.022
φ and ω scans	θ_max_ = 25.5°, θ_min_ = 1.8°
Absorption correction: multi-scan (*SADABS*; Bruker, 2004)	*h* = −6→6
*T*_min_ = 0.990, *T*_max_ = 0.994	*k* = −27→26
5177 measured reflections	*l* = −7→9

### Refinement

**Table:**

Refinement on *F*^2^	Secondary atom site location: difference Fourier map
Least-squares matrix: full	Hydrogen site location: inferred from neighbouring sites
*R*[*F*^2^ > 2σ(*F*^2^)] = 0.040	H-atom parameters not refined
*wR*(*F*^2^) = 0.137	*w* = 1/[σ^2^(*F*_o_^2^) + (0.092*P*)^2^ + 0.1151*P*] where *P* = (*F*_o_^2^ + 2*F*_c_^2^)/3
*S* = 1.00	(Δ/σ)_max_ = 0.001
1784 reflections	Δρ_max_ = 0.20 e Å^−^^3^
121 parameters	Δρ_min_ = −0.12 e Å^−^^3^
0 restraints	Extinction correction: *SHELXL97* (Sheldrick, 2008), Fc^*^=kFc[1+0.001xFc^2^λ^3^/sin(2θ)]^-1/4^
Primary atom site location: structure-invariant direct methods	Extinction coefficient: 0.016 (6)

### Special details

**Table:**

Geometry. All e.s.d.'s (except the e.s.d. in the dihedral angle between two l.s. planes) are estimated using the full covariance matrix. The cell e.s.d.'s are taken into account individually in the estimation of e.s.d.'s in distances, angles and torsion angles; correlations between e.s.d.'s in cell parameters are only used when they are defined by crystal symmetry. An approximate (isotropic) treatment of cell e.s.d.'s is used for estimating e.s.d.'s involving l.s. planes.
Refinement. Refinement of *F*^2^ against ALL reflections. The weighted *R*-factor *wR* and goodness of fit *S* are based on *F*^2^, conventional *R*-factors *R* are based on *F*, with *F* set to zero for negative *F*^2^. The threshold expression of *F*^2^ > σ(*F*^2^) is used only for calculating *R*-factors(gt) *etc*. and is not relevant to the choice of reflections for refinement. *R*-factors based on *F*^2^ are statistically about twice as large as those based on *F*, and *R*- factors based on ALL data will be even larger.

### Fractional atomic coordinates and isotropic or equivalent isotropic displacement parameters (Å^2^)

**Table:**

	*x*	*y*	*z*	*U*_iso_*/*U*_eq_	
C1	0.8254 (2)	0.15312 (5)	0.68372 (16)	0.0403 (3)	
C2	0.6652 (3)	0.20055 (6)	0.65592 (17)	0.0451 (4)	
H2	0.5490	0.2036	0.7183	0.054*	
C3	0.6795 (3)	0.24329 (7)	0.5346 (2)	0.0541 (4)	
H3	0.5698	0.2747	0.5180	0.065*	
C4	0.9907 (3)	0.19670 (8)	0.4672 (2)	0.0634 (5)	
H4	1.1040	0.1947	0.4022	0.076*	
C5	0.9919 (3)	0.15168 (7)	0.5851 (2)	0.0533 (4)	
H5	1.1034	0.1208	0.5980	0.064*	
C6	0.8303 (2)	0.10391 (6)	0.81421 (18)	0.0441 (4)	
C7	0.6972 (2)	0.11110 (6)	0.94016 (18)	0.0452 (4)	
H7	0.6012	0.1444	0.9377	0.054*	
C8	0.7118 (3)	0.06832 (6)	1.06560 (18)	0.0453 (4)	
H8	0.8147	0.0367	1.0632	0.054*	
C9	0.4227 (3)	0.11224 (8)	1.2011 (2)	0.0668 (5)	
H9A	0.5085	0.1490	1.2271	0.100*	
H9B	0.3519	0.1028	1.2948	0.100*	
H9C	0.2924	0.1150	1.0894	0.100*	
C10	0.6374 (3)	0.01980 (7)	1.3199 (2)	0.0641 (5)	
H10A	0.7505	−0.0083	1.2966	0.096*	
H10B	0.4815	0.0011	1.3112	0.096*	
H10C	0.7069	0.0355	1.4380	0.096*	
N1	0.8397 (3)	0.24245 (6)	0.44017 (18)	0.0617 (4)	
N2	0.5966 (2)	0.06690 (5)	1.18924 (16)	0.0494 (4)	
O1	0.9520 (2)	0.05981 (5)	0.80351 (16)	0.0677 (4)	

### Atomic displacement parameters (Å^2^)

**Table:**

	*U*^11^	*U*^22^	*U*^33^	*U*^12^	*U*^13^	*U*^23^
C1	0.0414 (7)	0.0407 (7)	0.0388 (7)	−0.0052 (5)	0.0120 (5)	−0.0024 (5)
C2	0.0477 (7)	0.0453 (8)	0.0433 (7)	−0.0010 (6)	0.0153 (6)	−0.0018 (6)
C3	0.0581 (9)	0.0476 (8)	0.0541 (9)	0.0035 (6)	0.0131 (7)	0.0068 (6)
C4	0.0583 (9)	0.0715 (11)	0.0699 (10)	0.0001 (8)	0.0337 (8)	0.0178 (8)
C5	0.0500 (8)	0.0541 (9)	0.0623 (9)	0.0035 (6)	0.0268 (7)	0.0092 (7)
C6	0.0480 (7)	0.0410 (7)	0.0460 (7)	0.0005 (5)	0.0184 (6)	0.0004 (6)
C7	0.0498 (7)	0.0429 (7)	0.0467 (7)	0.0020 (6)	0.0202 (6)	0.0009 (5)
C8	0.0499 (8)	0.0442 (8)	0.0460 (8)	−0.0016 (6)	0.0210 (6)	−0.0037 (6)
C9	0.0651 (10)	0.0810 (12)	0.0631 (10)	0.0146 (8)	0.0326 (8)	0.0008 (8)
C10	0.0914 (12)	0.0553 (9)	0.0583 (9)	−0.0050 (8)	0.0415 (9)	0.0036 (7)
N1	0.0633 (8)	0.0598 (8)	0.0646 (8)	−0.0018 (6)	0.0232 (7)	0.0176 (6)
N2	0.0576 (7)	0.0507 (7)	0.0476 (7)	0.0002 (5)	0.0274 (6)	0.0012 (5)
O1	0.0928 (9)	0.0519 (7)	0.0772 (8)	0.0237 (6)	0.0538 (7)	0.0171 (5)

### Geometric parameters (Å, °)

**Table:**

C1—C2	1.3846 (18)	C7—C8	1.3713 (19)
C1—C5	1.3842 (19)	C7—H7	0.9300
C1—C6	1.5148 (18)	C8—N2	1.3193 (17)
C2—C3	1.382 (2)	C8—H8	0.9300
C2—H2	0.9300	C9—N2	1.4476 (19)
C3—N1	1.329 (2)	C9—H9A	0.9600
C3—H3	0.9300	C9—H9B	0.9600
C4—N1	1.323 (2)	C9—H9C	0.9600
C4—C5	1.382 (2)	C10—N2	1.4555 (19)
C4—H4	0.9300	C10—H10A	0.9600
C5—H5	0.9300	C10—H10B	0.9600
C6—O1	1.2357 (16)	C10—H10C	0.9600
C6—C7	1.4180 (18)		
			
C2—C1—C5	116.72 (12)	C6—C7—H7	120.4
C2—C1—C6	124.44 (12)	N2—C8—C7	127.45 (13)
C5—C1—C6	118.84 (12)	N2—C8—H8	116.3
C1—C2—C3	119.39 (13)	C7—C8—H8	116.3
C1—C2—H2	120.3	N2—C9—H9A	109.5
C3—C2—H2	120.3	N2—C9—H9B	109.5
N1—C3—C2	124.31 (14)	H9A—C9—H9B	109.5
N1—C3—H3	117.8	N2—C9—H9C	109.5
C2—C3—H3	117.8	H9A—C9—H9C	109.5
N1—C4—C5	124.67 (14)	H9B—C9—H9C	109.5
N1—C4—H4	117.7	N2—C10—H10A	109.5
C5—C4—H4	117.7	N2—C10—H10B	109.5
C4—C5—C1	119.24 (14)	H10A—C10—H10B	109.5
C4—C5—H5	120.4	N2—C10—H10C	109.5
C1—C5—H5	120.4	H10A—C10—H10C	109.5
O1—C6—C7	124.21 (12)	H10B—C10—H10C	109.5
O1—C6—C1	117.19 (12)	C4—N1—C3	115.67 (13)
C7—C6—C1	118.60 (11)	C8—N2—C9	121.45 (12)
C8—C7—C6	119.15 (12)	C8—N2—C10	121.81 (13)
C8—C7—H7	120.4	C9—N2—C10	116.74 (12)
